# A Developed NK-92MI Cell Line with Siglec-7^neg^ Phenotype Exhibits High and Sustainable Cytotoxicity against Leukemia Cells

**DOI:** 10.3390/ijms19041073

**Published:** 2018-04-04

**Authors:** Chin-Han Huang, Yi-Jen Liao, Ting-Hsi Fan, Tzeon-Jye Chiou, Yen-Hsi Lin, Yuh-Ching Twu

**Affiliations:** 1 Department of Biotechnology and Laboratory Science in Medicine, School of Biomedical Science and Engineering, National Yang-Ming University, Taipei 112, Taiwan; pomelof76@gmail.com (C.-H.H.); pups0432@gmail.com (T.-H.F.); cathy501213@gmail.com (Y.-H.L.); 2 Department of Laboratory Medicine, Taipei Tzu Chi Hospital, Buddhist Tzu Chi Medical Foundation, New Taipei City 231, Taiwan; 3 School of Medical Laboratory Science and Biotechnology, College of Medical Science and Technology, Taipei Medical University, Taipei 112, Taiwan; yjliao@tmu.edu.tw; 4 Division of Transfusion Medicine, Department of Medicine, Taipei Veterans General Hospital, Taipei 112, Taiwan; tjchiou@vghtpe.gov.tw

**Keywords:** glycosylation, hypersialylation, immuno-surveillance, natural killer cell, Siglec-7

## Abstract

Altered sialic acid processing that leads to upregulation of cell surface sialylation is recognized as a key change in malignant tissue glycosylation. This cancer-associated hypersialylation directly impacts the signaling interactions between tumor cells and their surrounding microenvironment, especially the interactions mediated by immune cell surface sialic acid-binding immunoglobulin-like lectins (Siglecs) to relay inhibitory signals for cytotoxicity. First, we obtained a Siglec-7^neg^ NK-92MI cell line, NK-92MI-S7N, by separating a group of Siglec-7^neg^ cell population from an eight-month-long-term NK-92MI in vitro culture by fluorescence-activated cell sorting (FACS). The effect of Siglec-7 loss on NK-92MI-S7N cells was characterized by the cell morphology, proliferation, and cytotoxic activity via FACS, MTS assay, cytotoxic assay, and natural killer (NK) degranulation assay. We found the expression levels of Siglec-7 in NK-92MI were negatively correlated with NK cytotoxicity against leukemia cells. This NK-92MI-S7N cell not only shared very similar phenotypes with its parental cells but also possessed a high and sustainable killing activity. Furthermore, this Siglec-7^neg^ NK line was unexpectedly capable of eliminating a NK-92MI-resistant leukemia cell, THP-1, through enhancing the effector-target interaction. In this study, a NK cell line with high and sustainable cytotoxicity was established and this cell may provide a potential application in NK-based treatment for leukemia patients.

## 1. Introduction

Natural killer (NK) cells are a group of innate lymphocytes, comprising 5–20% of human peripheral blood lymphoid cells, and serve as an important constituent in host protection against viral infections and immune malignancy surveillance [1,2]. These cells can recognize and kill aberrant cells without preliminary antigen sensitization. NK activation and target recognition are determined by the inputs from a complex signal-integrating network that simultaneously receives and processes signals from two types of NK receptors, classified as activating and inhibitory, after engagement of their putative ligands on the target cell. The activating receptors include the family of natural cytotoxicity receptors (NCRs), DNAX accessory molecule-1 (DNAM-1), NK group 2 member D (NKG2D), and 2B4 [3,4,5]. Inhibitory receptors belong to either the superfamily of immunoglobulin or the c-type lectin receptors that mainly recognize major histocompatibility complex (MHC)-I molecules. NK-mediated immune function relies heavily on the dynamic signal balance from the inhibitory and activating receptors [4,6,7]. Appropriate interactions between NK inhibitory receptors and MHC-I molecules turn NK cells “off” and ensure self-tolerance. However, diminution or absence of cell surface MHC-I molecules, resulting from viral infections or tumor transformations, leads to a reduced overall inhibitory signal, thereby activating NK cells to eliminate those missing-self targets [8,9]. Specific killing of infected and transformed cells depends on several discrete steps that lead to polarization and exocytosis of lytic granules towards the target cells [10]. NK-target interaction, also known as conjugation formation, is the first key step for NK cell cytotoxicity [11]. Engagement of NK cell surface integrin lymphocyte function-associated antigen 1 (LFA-1), a heterodimer of CD11a and CD18, by its ligand intercellular adhesion molecule 1 (ICAM-1) on target cells establishes a stable adhesion of NK cells to their target cells and is sufficient to induce the polarization of lytic granules in resting NK cells [12,13,14]. Then, NK-cell cytotoxicity is achieved by degranulation of these accumulated granules toward the direction of the engaged target.

Tumor/leukemia cells are known for their ability to escape host immunity. Malignancy can even develop in a host with a fully functional immune system, which is known as the cancer immunoediting concept [15]. During tumorigenesis and leukemogenesis, mutations and epigenetic changes not only affect the protein composition, but also induce profound changes in glycosylation [16,17,18]. One of the hallmarks of such altered glycosylation is the upregulation of terminal sialic acids on secreted or cell surface glycoconjugates. Upregulation of cell surface sialylation has been implicated in complement-mediated tumor cell killing, enhancement of tumor-related inflammation, and especially cancer progression where upregulation of selectin ligands influences metastasis colonization [19,20]. Moreover, recent experimental findings suggest that interactions between hypersialylated ligands on tumor cells and NK cell surface immunomodulatory sialic acid-binding immunoglobulin-like lectins (Siglecs) contribute to cancer immunosurveillance and cancer-associated immune suppression [21,22,23,24], implying the involvement of the NK cell surface Siglecs in cytotoxicity. Restrictedly expressed only on certain cell types in humans, there are 15 functional members in the Siglec family featuring a N-terminal V-set Ig-like domain, which characteristically mediates carbohydrate recognition for signal transduction, followed by a varying number of C2-set Ig-like domains [25,26]. For most Siglecs, recognition of their ligands can initiate cellular signaling through immunoreceptor tyrosine-based inhibitory motifs (ITIMs) at their cytoplasmic tails. By function, Siglecs with ITIMs are able to recruit phosphatase, and are therefore referred to as inhibitory-type Siglecs [27,28]. Siglec-7 (also termed p75 adhesion inhibitory receptor molecule 1, p75/AIRM-1, CDw328) is expressed on most human peripheral blood NK cells, monocytes, basophils, and mast cells [29,30,31,32]. Siglec-7 contains three extracellular immunoglobulin-like domains, a transmembrane region, and a cytoplasmic tail with two tyrosine residues embodied in ITIM-like motifs [30]. Of the other Siglecs expressed by NK cells, Siglec-9 exists in both T cells and basophil cells, and is expressed by 50% of NK cells [33,34]. Revealed by recent studies, role of Siglec-7 is emerging as an important regulator in the immune response. In redirected killing assay, Siglec-7 functions as a NK inhibitory receptor for its ability to inhibit human NK cell cytotoxicity against murine P815 target cells [29]. Also, sialic acid moieties of tumor cell surfaces can be recognized by Siglec-7 to negatively modulate NK cell killing activity, likely leading to escape from NK cell cytotoxicity [35]. Interestingly, increased expression of sialylated ligands observed on tumor cell surface inhibits NK cell activation through the engagement with Siglec-7 [21,24]. On the other hand, decreased expression of NK Siglec-7 has been consistently observed during cancer treatment and such low levels last until the end of treatment [36,37]. These results suggest loss of Siglec-7 signaling favors NK cell activation. With the ability to eliminate transformed and tumor/leukemia cells, the NK cells have been considered as an alternative therapy to the conventional chemo- or radioactive treatment. Among NK cell-based therapies, a developed NK cell line, NK-92, has been applied in clinical trials [38,39,40]. However, the sustainability of the infused NK-92 and intensity of cytotoxicity in the treatment protocol still have room for refinement, such as by manipulating cytotoxic activity through enhancing the activating receptor-mediated signals, or eliminating/reducing the inhibitory signals to prolong or enhance its effect.

In this study, we initially found that the cytotoxicity of NK-92MI cells declined over the course of long-term in vitro culture. We hypothesized that there is an important factor or more involved in regulating such NK function along the culture progress and we discovered a correlation between the presence of cell surface Siglec-7 and NK-92MI cytotoxicity. Hence, we established a Siglec-7^neg^ NK cell model (NK-92MI-S7N), derived from NK-92MI, to investigate Siglec-7 effect on NK cytotoxicity, especially on eliminating leukemia cells. Our findings demonstrated that the NK-92MI cells without the presence of Siglec-7 shared very similar phenotypes with parental NK-92MI in cell morphology, expressions of NK activating and inhibitory receptors, and cytotoxic-related proteins and cytokines. However, they possessed higher ICAM-1 binding avidity and affinity as well as high and sustainable cytotoxic activity that was able to eliminate a NK-92MI-resistant cell. With the development of this NK model, there may be an applicational potential for some NK-92-based immunotherapies.

## 2. Results

### 2.1. Presence of Increased Cell Surface Siglec-7 Expression Correlated with Significant Reduction in Cytotoxicity of the NK-92MI Cell

**Figure 1:**
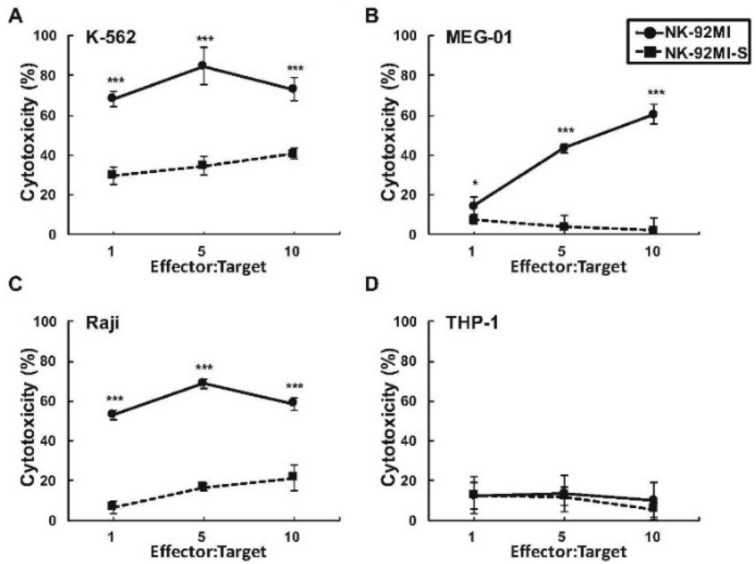
Comparisons of the cytotoxicity between NK-92MI and NK-92MI-S against four leukemia cell lines. K-562 (**A**); MEG-01 (**B**); Raji (**C**); and THP-1 (**D**) were tested for their sensitivity to NK lysis by using Non-Radioactive Cytotoxicity Assay at different effector:target (E:T) ratios, as indicated. The results were representative of three experiments. Data are presented as mean ± SD of triplicates. * *p* < 0.05, *** *p* < 0.001, Student’s *t* test.

Although NK-92MI cell is known for its application in clinical trials as the first NK cell line used in NK-mediated immunotherapy against cancer and leukemia [38,39,40], the details of its regulatory mechanism for such cytotoxicity response are still under investigation. More understanding of this mechanism, will greatly benefit current NK-based treatments for cancer/leukemia and may even lead to possible manipulations on cytotoxicity for the desired treatments. Initially when NK-92MI cells were cultured in vitro, these cells morphologically formed aggregations, in which, interestingly, size positively corresponded to their cytotoxicity. Importantly, the gradual loss of killing activity of NK-92MI cells over the course of in vitro culture was also observed, suggesting killing function/activity of cultured NK-92MI cells could be modulated by unknown regulatory factor(s) over time. To compare the difference in cytotoxicity over time, the smaller aggregation-forming cells obtained from higher passage numbers (3-month-long-term culture), designated as NK-92MI-S, were compared to parental NK-92MI. By non-radioactive cytotoxicity assay, the killing activities were significantly lower in the NK-92MI-S against three selected leukemia cell lines, at effector:target (E:T) ratios from 1:1 to 10:1, as opposed to those observed in parental NK-92MI (Figure 1A–C). On the other hand, consistent with our previous findings on the NK-92MI-resistant THP-1 cell line, there was no noticeable difference in cytotoxicity against this cell between two NK lines (Figure 1D) [41].

**Figure 2:**
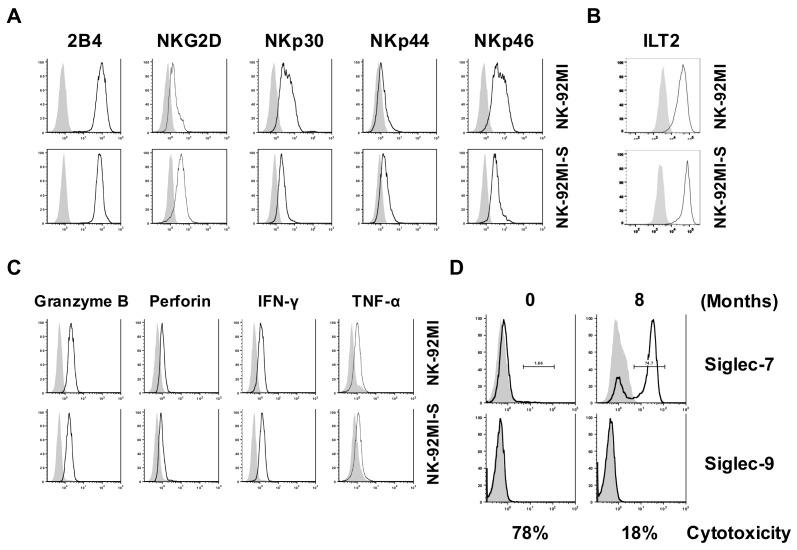
Comparison of NK cell properties between NK-92MI and NK-92MI-S cells. Flow cytometric analyses for the presence of NK activating receptors (**A**); inhibitory receptor (**B**); cytotoxic-related proteins (**C**); and inhibitory Siglec receptors (**D**) of the NK cells. The open and shaded area represented the results obtained from cells incubated with indicated antibodies and isotype control. The results shown were representative of three independent experiments. The numbers shown in (**D**) represent the cytotoxicity as a percentage against Raji by using CytoTox96 Non-Radioactive Cytotoxicity Assay Kit.

To investigate whether observed lower cytotoxicity in NK-92MI-S was influenced by the change in the expressions of surface activating receptors, inhibitory receptors, production of cytotoxic proteins in the cytotoxic granules, or cytokines of the NK cells, we examined the expressions of 2B4, NKG2D, NKp30, NKp44, NKp46, ILT2, programmed death 1 (PD-1), granzyme B, perforin, IFN-γ, and TNF-α. Unexpectedly, the parental and NK-92MI-S cells shared similar expression levels for most of the examined factors, except for slightly higher expressions of NKp30 and NKp46 observed in the highly cytotoxic parental cells (Figure 2A). As initiation of killing activity for NK cells depends on the net overall signaling received from both activating and inhibitory receptors before releasing cytotoxic-related proteins, we investigated the expressions of two key inhibitory receptors, ILT2 and PD-1, as well as cytotoxic proteins. The results showed that there was no noticeable difference among levels of ILT2, PD-1, and cytotoxic proteins between parental and NK-92MI-S cells (Figure 2B,C).These results, suggested that the examined factors involved in cytotoxic-related receptors and proteins did not contribute to the lower cytotoxicity found in NK-92MI-S.

Next, we studied the expressions of tumor-associated carbohydrate antigens (TACA)-related inhibitory receptors, Siglec-7 and Siglec-9, on the NK-92MI and -S cells. We found that the Siglec-7 expression on the cultured NK-92MI cells gradually increased over the course of the in vitro culture time but observed no such expression pattern on Siglec-9 (Figure 2D). Our results showed a correlation between the change in Siglec-7 expression and the decrease in NK cytotoxicity along the culture time course (Figure 1 and Figure 2D). Interestingly, a group of about 25% NK-92MI-S cells still exhibited an undetectable Siglec-7 phenotype when cultured for more than 8 months and could still maintain such phenotype in culture for more than 16 months (Figure 2D and not shown results). Based on this finding, we hypothesized that the low cytotoxicity observed in NK-92MI-S cells resulted from the upregulation of cell surface Siglec-7 that subsequently enhanced the overall inhibitory signal for the killing activity.

### 2.2. The Establishment of a Siglec-7^neg^ NK Cell Model

**Figure 3:**
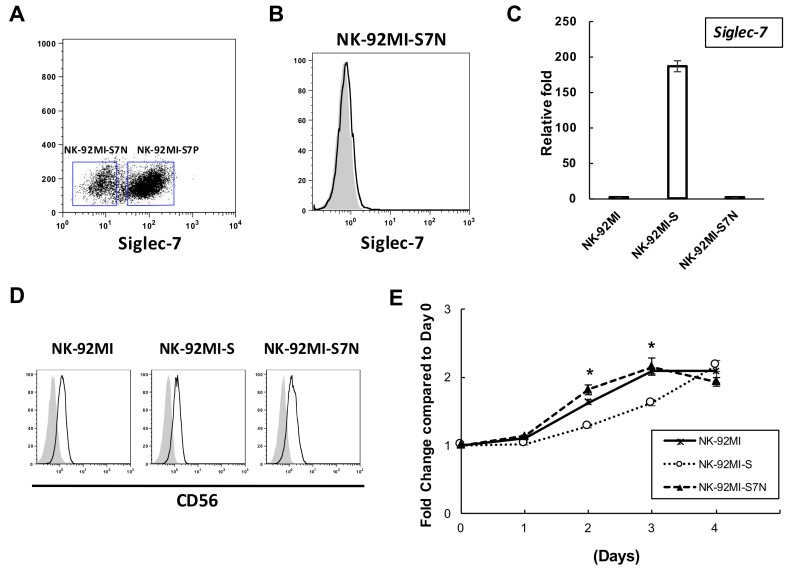
Establishment of the NK-92MI-S7N cell. (**A**) Gating strategy and sorting for Siglec-7^pos^ and Siglec-7^neg^ subgroups from the 8-month-long-term cultured NK-92MI cells; (**B**) Representative flow cytometric staining pattern for Siglec-7 on the sorted NK-92MI-S7N cells. The open and shaded area represented the results obtained from cells incubated with anti-Siglec-7 antibodies and isotype control, respectively; (**C**) Real-time PCR analysis of *Siglec-7* transcripts in NK-92MI, -S, and -S7N cells. *Siglec-7* expression was related to parental control and the expressions were normalized to *GAPDH*. The data was presented as the mean ± SD of three independent experiments; (**D**) Flow cytometric analysis of NK marker CD56 in NK-92MI, -S, and -S7N cells. The open and shaded area represented the results obtained from cells incubated with anti-CD56 antibodies and isotype control, respectively; (**E**) Cell growth assay on three NK cells. Fold of growth was normalized to day 0. *n* = 4 independent biological replicas with three technical replicas each. * *p* < 0.05 at Day 2 and Day 3 among NK-92MI-S and two others, NK-92MI and -S7N.

Given the correlation between Siglec-7 expression and NK cytotoxicity, and the lack of Siglec-7 observed in a subgroup of the long-term NK-92MI-S culture, we asked whether this particular subset of NK-92MI-S cells with the Siglec-7^neg^ phenotype can be established as a unique cell line in which its cytotoxicity can be sustainable over time as the consequence of loss of Siglec-7 expression. To achieve this goal, a bulk 8 month-long-term cultured NK-92MI-S cells, based on the Siglec-7 expression, were stained and sorted. Cells with and without Siglec-7 expression were collected and designated as NK-92MI-S7P and NK-92MI-S7N, respectively (Figure 3A). Interestingly, the purified NK-92MI-S7P cells failed to survive for more than 2 weeks of in vitro culture from three independent attempts. In contrast to NK-92MI-S7P, purified NK-92MI-S7N proliferated normally and morphologically formed large aggregations, as the parental cells did. By FACS analysis, these NK-92MI-S7N cells still maintained Siglec-7^neg^ phenotype after long-term culture over one year (Figure 3B). In addition to the surface Siglec-7 expression, the *Siglec-7* transcript in NK-92MI-S7N cells was examined by quantitative RT-PCR and we found that its level was as low as that in the parental cells as opposed to the high expression in NK-92MI-S (Figure 3C). Additionally, this established cell exhibited a similar level of a NK marker, CD56, in comparison with the parental NK-92MI and -S cells (Figure 3D). To characterize the proliferation rate of NK-92MI-S7N cells, we performed the MTS assay and found that both the NK-92MI and -S7N displayed very similar proliferation rates where both rates were slightly faster than that of NK-92MI-S cells on Day 2 and Day 3 (Figure 3E). In this section, we purified and established a unique Siglec-7^neg^ NK-92MI-S7N stable cell line to elucidate how loss of Siglec-7 affected the behavior of NK-92MI cytotoxicity.

### 2.3. The Developed NK-92MI-S7N Cell Possessed a High and Sustainable Killing Activity against Leukemia Cells

**Figure 4:**
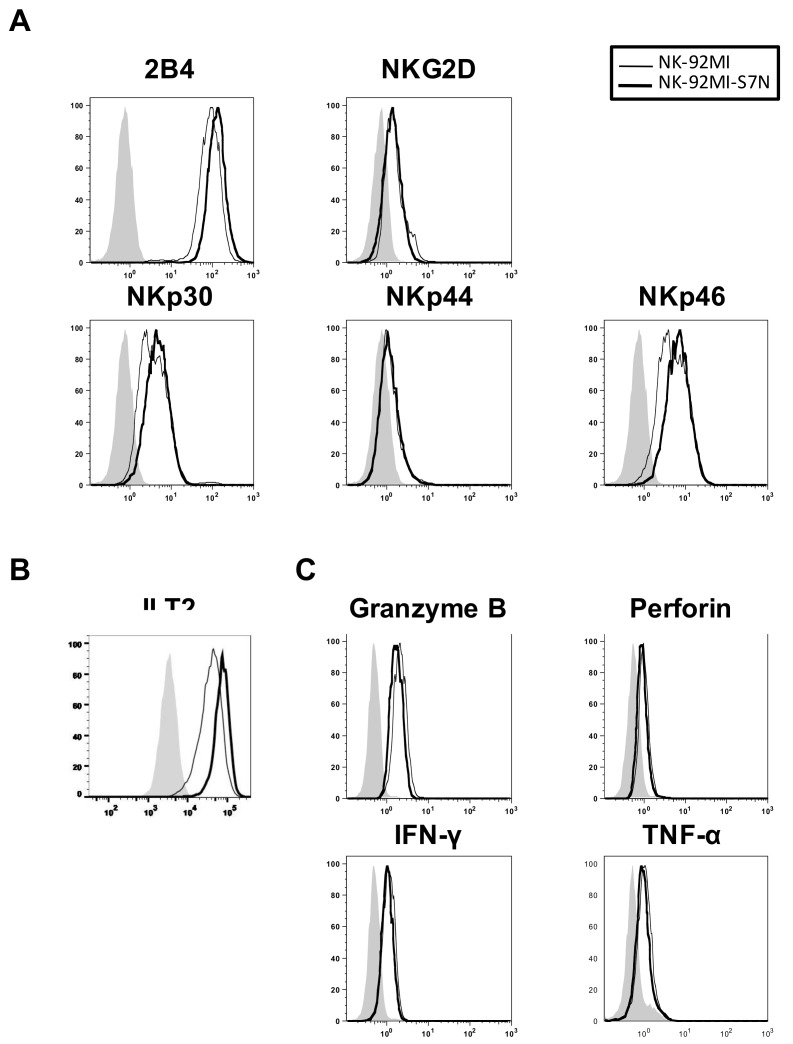
Characterizations of the NK-92MI-S7N phenotypes. Flow cytometric comparisons for the presence of NK activating receptors (**A**); inhibitory receptor (**B**); and cytotoxic-related proteins (**C**) between the NK-92MI and -S7N cells. The shaded area represented the results obtained from cells incubated with isotype control. And thin and heavy lines represented the results obtained from designated antibodies against NK-92MI and -S7N, respectively. The results shown were representative of three independent experiments.

First, we compared developed NK-92MI-S7N with the parental cells for whether loss of Siglec-7 affected factors involved in NK cytotoxicity, including the expressions of both NK activating and inhibitory receptors, and the levels of cytotoxic-related proteins. We performed the FACS analysis and found that, compared to the parental cells, NK-92MI-S7N cells possessed slightly higher levels of 2B4, NKp30, NKp46, and ILT2, but expressed similar levels of NKG2D, NKp44, and PD-1 (Figure 4A,B and not shown results). On the other hand, the amount of cytotoxic-related proteins and cytokines displayed no noticeable difference between these two NK cell lines (Figure 4C).

**Figure 5:**
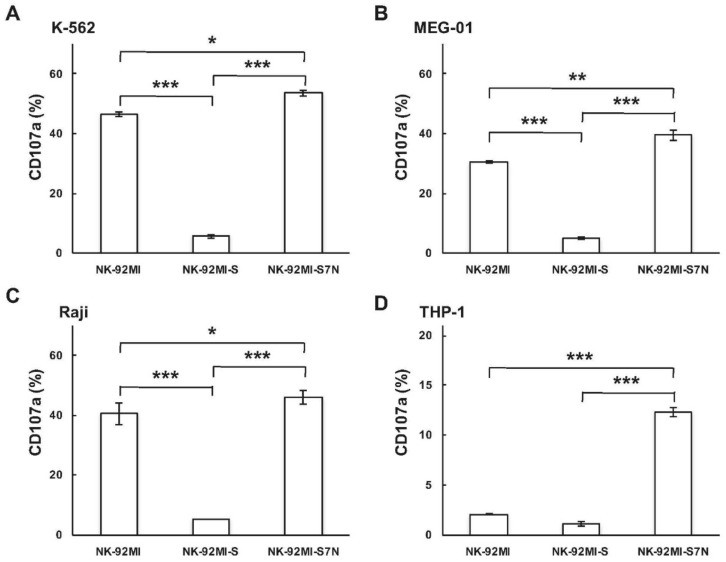
Effect of the inhibitory receptor Siglec-7 on the NK degranulation activity. CD107a degranulation in NK-92MI, -S, and -S7N were examined by incubations of K-562 (**A**); MEG-01 (**B**); Raji (**C**), and THP-1 (**D**) with fluorescence-labeled NK cells at a 1:1 ratio of effector:target. Results were presented as mean ± SD of triplicates (* *p* < 0.05, **p < 0.01 *** *p* < 0.001). The results were representative of three independent experiments.

Next, we characterized how the degranulation activity of developed NK-92MI-S7N cells was, by CD107a assay, among these three NK-92MI cell lines, parental, -S, and -S7N since the presence of cell surface CD107a molecule served as a read-out mark of NK cell degranulation and cytotoxicity [42]. Consistent with previous results obtained from non-radioactive cytotoxicity, the parental NK-92MI displayed higher cytotoxicity than that of the NK-92MI-S against K-562, MEG-01, and Raji, but was incapable of killing NK-resistant THP-1 cells as was NK-92MI-S (Figure 1 and Figure 5). However, surprisingly, we found that NK-92MI-S7N not only possessed functional cytotoxic activity but also exhibited higher degranulation activity than parental NK-92MI did against all selected target leukemia cells (Figure 5). More importantly, the NK92MI-S7N cells were able to eliminate NK-92MI-resistant THP-1cells, as evidenced by the significantly high degranulation activity in the NK-92MI-S7N group (Figure 5D). To further confirm the role of Siglec-7 inhibitory signaling in NK killing activity assay, we examined how the susceptibility of THP-1 cells, while pre-treated with neuraminidase to remove cell surface sialic acid ligands, was affected in three NK cells. We found that removal of sialic acids on THP-1 cells enhanced the sensitivity to NK-92MI killing by around 50%, but no such change was observed in the NK-92MI-S7N cell (Figure S1). Based on these results, we showed that target cell sensitivity could be greatly enhanced by inhibiting Siglec-7 signaling and our developed NK-92MI-S7N, without Siglec-7 expression, functioned normally and possessed a high cytotoxicity phenotype against tested leukemia cells, even the NK-resistant THP-1 cells.

**Figure 6:**
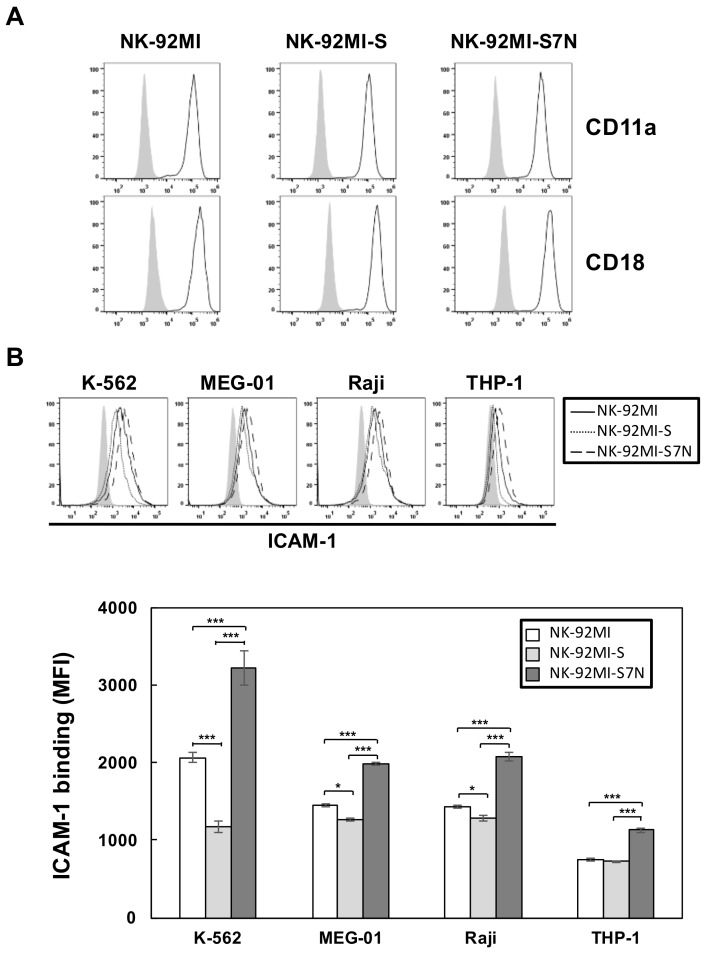
Characterization of LFA-1 binding activity in three NK cell lines. (**A**) Flow cytometric analysis for the presence of CD11a and CD18 on the NK-92MI, -S, and -S7N cells, as indicated. The shaded and open area represented the results obtained from cells incubated with isotype control and indicated antibodies, respectively. The results shown are representative of three independent experiments; (**B**) Measurements of LFA-1 binding in three NK cell lines against four different leukemia cells. Indicated NK cells were stimulated, by 2B4 activation, against designated leukemia cells at a 1:1 ratio of NK:target. LFA-1 binding was measured by the presence of ICAM-1-Fc complexes (upper panel). The shaded area represented the results from cells incubated with antibody control (CD99-Fc). The solid, dashed, and long dashed lines represented the results from NK-92MI, -S, and -S7N, respectively. The mean fluorescence intensity (MFI) of ICAM-1 binding for each NK cell was summarized (lower panel). Values represented means of three independent experiments ± SD (* *p* < 0.05, *** *p* < 0.001). The results were representative of three independent experiments.

Given that the effector-target interaction is a critical step for efficient NK cytotoxicity, during which the LFA-1 binding activity plays a major role to stabilize the interaction with ICAM-1 located on the target cells, we next investigated whether the difference in degranulation function among NK-92MI-S7N and two others resulted from the LFA-1 binding. As LFA-1 binding affinity is affected by both cell surface expressions and conformation upon the engagement of target cells, we first compared the expression levels of two LFA-1 subunits, CD11a and CD18, among three NK lines. Results showed that these three NK lines possessed the same high, but no noticeably different, expressions of both CD11a and CD18 (Figure 6A). Next, we examined the LFA-1 binding among these three NK cells by performing a ligand complex-based adhesion assay (LC-AA), through 2B4-dependent NK activation, to evaluate the overall binding capacity, the affinity and avidity of LFA-1 [43,44]. We found that NK-92MI-S7N cells possessed highest LFA-1 binding against four different leukemia lines as strongly evidenced by the ICAM-1-Fc complex binding in the LC-AA, followed by the parental NK-92MI with intermediate staining, and last by the NK-92MI-S with least active ICAM-1-Fc complex binding on their surface (Figure 6B). This result demonstrated that the central first step of effector-target interaction, mediated by LFA-1, could be greatly influenced by the presence of cell surface Siglec-7, and suggesting Siglec-7 loss affected the very upstream of the entire NK cytotoxicity process leading to the downstream response of enhanced degranulation activity against leukemia cells (Figure 5).

**Figure 7:**
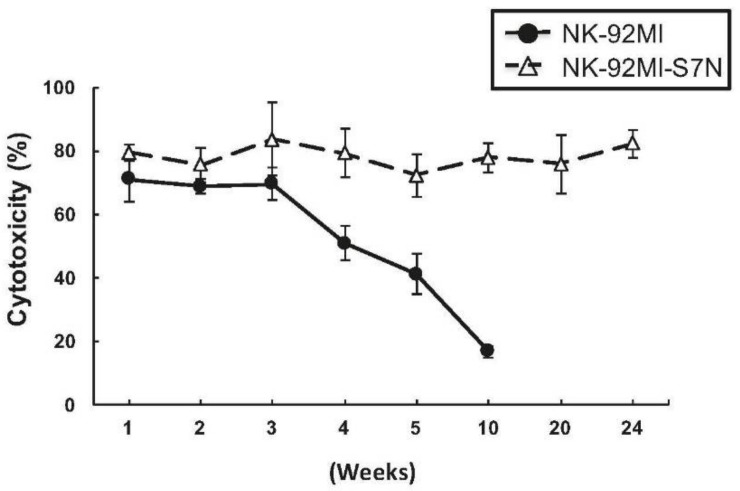
The Siglec-7^neg^ NK cells possessed a phenotype of prolonged cytotoxicity. Over a period of time, cytotoxicity measurements for NK-92MI and -S7N against Raji cells was conducted, as indicated, by using Non-Radioactive Cytotoxicity Assay at E:T ratio 5:1. Data was presented as mean ± SD of triplicates. The results were representative of three experiments.

Last, we monitored the cytotoxicity change of NK-92MI-S7N and the parental cells over the course of 24-week in vitro culture. We observed that the killing activity of NK-92MI-S7N was sustainable at the same level after 24 weeks, as opposed to that of the parental NK-92MI where activity started to decline to 50% in just 5 weeks and less than 20% in 10 weeks (Figure 7). This result demonstrated that this established high cytotoxic NK-92MI-S7N cells not only were capable of killing leukemia cells, including resistant THP-I, but also maintained its cytotoxicity over an extended period of time. 

## 3. Discussion

Recently, manipulation of inhibitory receptors has been exploited in the context of both innate and adaptive immunity to enhance antitumor activity. For example, blockade of the T cell checkpoint inhibitor, CTLA-4 and PD-1, has been shown to stimulate more durable clinical responses in a limited number of patients [45,46]. Using a similar strategy, in this study we established an effector NK cell line, NK-92MI-S7N, with Siglec7^neg^ phenotype that was not able to relay the inhibitory signaling from those hypersialylated ligands and this phenotype served as a useful manipulation in anti-tumor/leukemia immunity and subsequent application. Earlier studies have demonstrated that Siglec-7 acts as an inhibitory role in antibody-dependent cytotoxicity to GD3-expressing targets, T cell receptor-dependent signaling, and Ca^2+^ flux to attenuate cellular signals, suggesting its broad inhibitory effect during immune response [29,35,47]. Our results showed that loss of Siglec-7 expression did not compromise the NK property in this cell line as the NK-92MI-S7N cell model functioned normally in various aspects, including the cell morphology, proliferation, and expressions of NK marker, activating receptors, inhibitory receptors, and cytotoxic-related proteins, as the parental NK-92MI cells did (Figure 3, Figure 4 and Figure 6). Moreover, while examining the cytotoxicity response of NK-92MI-S7N cells, we observed an overall better degranulation activity than that of the parental cells against four different leukemia cell lines (Figure 5). Surprisingly, this elevated killing activity of NK-92MI-S7N was even capable of killing a NK-92MI-resistant cell, THP-1, resulting from the increased cell-cell interaction between Siglec-7^neg^ NK and THP-1 cells through LFA-1-ICAM-1 recognition (Figure 5D Figure 6B and not shown data).

In this study, NK-92MI-S7N was obtained from the long-term culture of NK-92MI-S. The mechanism for this Siglec-7^neg^ phenotype is unknown, possibly from *Siglec-7* mutation(s) or gene inactivation as spontaneous mutation(s) could occur over the course of long-term culture. Excluding mutation(s) directly responsible for the change of Siglec-7 expression, other natural occurring mutations were not likely to be the contributing factor for the high and sustainable cytotoxic features observed in NK-92MI-S7N but not in NK-92MI-S7P. This is because mutation(s), dominant or not, should already be evenly distributed in the whole cell population after long-term culture. Their effect on cytotoxicity should be global and not just be restricted to NK-92MI-S7N, which was separated from the whole population only by the presence of Siglec-7 (Figure 3A). Additionally, this notion of enhanced cell sensitivity through Siglec-7 loss could be further supported by the result that THP-1 cell became more sensitive to NK-92MI and NK-92MI-S targeting once the presence of sialic acids on its cell surface was reduced by neuraminidase treatment (Figure S1). After long-term culture of the NK-92MI cells, in addition to the expression of surface Siglec-7 receptor, the *Siglec-7* transcripts were measured with up to a 160-fold increase in NK-92MI-S as opposed to those of other two cell lines (Figure 3C). These upregulated *Siglec-7* transcripts in the NK-92MI-S cells can result from activation of transcription regulator(s), epigenetic modifications, such as DNA methylation on its regulatory region, or the decrease of microRNA targeting on the *Siglec-7* transcripts. Hence, future characterizations to identify the involved regulatory mechanism(s) of *Siglec-7* activation and Siglec-7-related effector-target interaction/killing could greatly increase our understanding about its relation with NK cytotoxicity.

The hypersialylated glycans on the tumor or leukemia cells have been shown, in different types of cancer including melanoma, breast, prostate, and non-small cell lung cancers, to inhibit NK function in vitro and in vivo through the interactions with these Siglec receptors, including Siglec-7 and Siglec-9 [21,22,23,48,49,50]. Functioning as an inhibitory receptor expressed on NK cells, Siglec-9, another member of CD33-related Siglecs, has been demonstrated to play a negative role for reactive oxygen species (ROS) production on neutrophils to suppress anti-tumor activity [51]. In macrophage, both Siglec-7 and -9 were upregulated along the macrophage colony-stimulating factor (M-CSF)- and granulocyte-macrophage colony stimulating factor (GM-CSF)-induced macrophage differentiation, but Siglec-7 and -9 were differently expressed when responding to LPS/IFN-γ, suggesting their regulation might be under different pathways [52]. Interestingly, in the NK-92MI model, Siglec-9 expression pattern was different from that of Siglec-7 as evidenced by not being detected among these three NK lines by FACS analysis (Figure 2D and not shown data) [21]. In combination with these findings that the interaction between Siglec-7 receptor and ligand influences NK cell-dependent tumor immunosurveillance, it is likely that the outcome of using allogeneic KIR-mismatched hematopoietic stem cell transplantation in the clinical setting of leukemia could be further enhanced by just blocking cell surface Siglec-7, but not necessarily Siglec-9, on donor cells.

Expression of cell surface glycans is known to be involved in many key regulatory functions during the immune response, like correlation with the cell transformation into the tumor and leukemia as well as the escape from host immunity, such as from cytotoxic T- and NK-mediated surveillance [41,53]. Currently, it is still not clear how expression of Siglec-7 is regulated in both therapeutic NK-92MI and normal human NK cells. However, based on our results, expression of Siglec-7 was upregulated in NK-92MI over the course of in vitro culture as compared to being downregulated during human NK maturation (from CD56^bright^ to CD56^dim^) [54]. Such expression pattern of Siglec-7 on NK-92MI will be a challenge for some NK-92MI-based clinical tumor/leukemia treatments as these malignant cells can escape from immuno-surveillance by over-expressing surface sialylated glycans to enhance overall inhibitory signaling. Our developed new NK-92MI cell model, NK-92MI-S7N, which is less responsive to hypersialylated glycans, can overcome this problem of tumor cells with aberrant sialylation.

For NK-based therapy against malignant diseases, patients currently need frequent NK transplantations for treatment to be effective. However, such efficacy could be greatly enhanced if the cytotoxic activity of the transplanted NK cells can be maintained and sustained over time, like our NK-92MI-S7N cell system with long-lasting high cytotoxicity for over 12 months (Figure 7 and not shown results). Taken together, in this study, this is the first time we have shown that NK cells with Siglec-7^neg^ phenotype possess a high and sustainable killing activity and have the capability to eliminate a NK-resistant leukemia cell and hypersialylated tumor cells, two important characteristics that could potentially improve current NK-based tumor/leukemia therapy.

## 4. Materials and Methods

### 4.1. Cell Culture

Human NK cell line, NK-92MI, and four leukemia cells, K-562, MEG-01, Raji, and THP-1 were purchased from Bioresource Collection and Research Center (Hsinchu, Taiwan). NK-92MI cells were grown in α-MEM medium supplemented with 20% fetal bovine serum, 12.5% horse serum, 50 units/mL of penicillin, and 50 μg/mL of streptomycin in a humidified incubator with 5% CO_2_ at 37 °C. Other leukemia cell lines were grown in 90% RPMI 1640 medium with 10% fetal bovine serum, 50 units/mL of penicillin, and 50 μg/mL of streptomycin. All reagents were purchased from Invitrogen (Carlsbad, CA, USA).

### 4.2. Non-Radioactive Cytotoxicity Assay 

The killing activity of NK cell against the leukemia target was performed by using CytoTox96 Non-Radioactive Cytotoxicity Assay (Promega, Madison, WI, USA). Briefly, the NK-92MI cells (effector) were co-cultured with target cells with varying ratios of effector to target for 4 h at 37 °C. The release of lactate dehydrogenase from lysed target cells was then measured. All assays were performed in triplicate. Percent cytotoxicity = (experimental lactate dehydrogenase release − effector spontaneous release − target spontaneous release)/(target maximum release − target spontaneous release) × 100%.

### 4.3. Flow Cytometric Analysis

The expression of cell surface-bound receptors and intracellular proteins of NK cells was assayed by flow cytometry, with the commercially available antibodies against 2B4, granzyme B, IFN-γ, NKG2D, NKp30, NKp44, NKp46, perforin, Siglec-7 (eBioscience, San Diego, CA, USA), CD11a, CD18, ILT2 (BioLegend, San Diego, CA, USA), Siglec-9, and TNF-α (BD Biosciences, San Joes, CA, USA) with fluorescence-conjugation. For intracellular staining of cytokines, GolgiPlug (BD Biosciences) was added to block cytokine secretion. Samples were subjected to Cytomic FC500 or CytoFLEX (Beckman Coulter, Fullerton, CA, USA) flow cytometry equipment and results were analyzed using FlowJo software (FlowJo v10, LLC, Portland, OR, USA) or CytExpert (CytExpert 2.1, Beckman Coulter).

### 4.4. Establishment of Siglec7^neg^ NK-92MI (NK-92MI-S7N) Cells

The 8-month-long-term cultured NK-92MI cells were first labeled with phycoerythrin (PE)-conjugated anti-Siglec-7, and cell population was sorted and separated by S3e Cell sorter (Bio-Rad; Hercules, CA, USA). The resulting cells, NK-92MI-S7P (Siglec-7^pos^) and NK-92MI-S7N (Siglec-7^neg^) were cultured under the same conditions as the parental cells described above. FACS analysis was performed to re-confirm the Siglec-7 expression of these two sorted cell populations. The established Siglec-7^neg^ cells were used for further experiments.

### 4.5. Quantification of Siglec-7 Transcripts

Total RNA isolation and the first-strand cDNA synthesis were prepared as previously described [55]. Target transcripts in the cDNA samples were quantified using TaqMan gene expression assay kits (Applied Biosystems, Foster City, CA, USA) with *Siglec-7* (Hs01100854_m1) and *GAPDH* (Hs99999905_m1) as primers and probes. For each target, PCR was performed and detected with the StepOnePlus Real-Time PCR system (Applied Biosystems).

### 4.6. Proliferation Assay

The proliferation of NK-92MI, -S, and -S7N cells was evaluated using a commercial reagent, Cell Titer 96 Aqueous One Solution Cell Proliferation Assay (Promega), including 3-(4,5-dimethylthiazol-2-yl)-5-(3-carboxymethoxyphenyl)-2-(4-sulfophenyl)-2H tetrazolium (MTS) and phenazine methsulfate (PMS; Sigma-Aldrich, St. Louis, MO, USA). The cells were seeded onto a 96-well plate at a density of 1.2 × 10^5^ cells/mL and were then cultured in a CO_2_ incubator at 37 °C for 4 days. At each 24-h interval, cell culture was changed with fresh medium, added with MTS/PMS, and incubated at 37 °C for 90 min before the measurement of proliferation by the absorbance reading at 490 nm using a microplate reader (TECAN, Mannedorf, Switzerland).

### 4.7. NK Degranulation Assay (CD107a Assay)

Far red fluorescent (CellVue Claret Far Red Fluorescent Cell Linker Kit; Sigma-Aldrich)-labeled NK cells were incubated with equal number of target cells at 37 °C for 60 min. Following the culture, cell mixture was stained with PE-conjugated CD107a antibody (eBioscience). Samples were subjected to CytoFLEX flow cytometry and results were analyzed using CytExpert. To determine the CD107a expression of NK cells, the CD107a positive rate of fluorescent-labeled NK cells was analyzed. 

### 4.8. Ligand Complex-Based Adhesion Assay (LC-AA) 

The LC-AA was performed according to a modified version of previous research [43,44]. Basic buffer for all incubation is PBS containing 0.5% BSA, supplemented with or without cations (1 mM CaCl_2_ and 2 mM MgCl_2_). The ICAM-1-Fc complexes were prepared by mixing 50 μg/mL recombinant human ICAM-1-Fc chimera (R&D Systems, Minneapolis, MN, USA) and F(ab)_2_ fragment of goat anti-human Fcγ fragment specific (40 μg/mL PE-labeled; Jason Immuno-Research, West Grove, PA, USA) in buffer without cations for 16 h at 4 °C. As a negative control, CD99-Fc was used instead of ICAM-1-Fc. For the target activation for three examined NK cell lines, each was incubated with 5(6)-Carboxyfluorescein diacetate *N*-succinimidyl ester (CFSE; Sigma-Aldrich)-labeled target leukemia cells, at E:T ratio of 1:1 for 30 min at 37 °C. The cell mixture was incubated for 10 min with 1 μg/mL of 2B4 (Invitrogen; clone C1.7) or isotype control. After washing, cells were resuspended in buffer for addition of ICAM-1-Fc complexes (dilution 1:20) before being transferred to 37 °C incubator for 15 min. Cells were fixed by adding warm (37 °C) paraformaldehyde to a final concentration of 2%. After 5 min, fixation was stop by adding ice-cold buffer, then the cells were pelleted and analyzed on CytoFLEX and FlowJo software. The LFA-1 binding capacity was detected for the bound ICAM-1-PE on NK cell lines (CFSE^neg^ population).

## Supplementary Materials

The following are available online at http://www.mdpi.com/1422-0067/19/4/1073/s1.
