# Supplementary material for: A Developed NK-92MI Cell Line with Siglec-7neg Phenotype Exhibits High and Sustainable Cytotoxicity against Leukemia Cells
Source: Int J Mol Sci. 2018 Apr 4;19(4):1073. doi: 10.3390/ijms19041073 (PMC5979288; doi:10.3390/ijms19041073)
Supplement: Supplementary file 1 [file ijms-19-01073-s001.pdf]

Supplementary Data

Figure S1

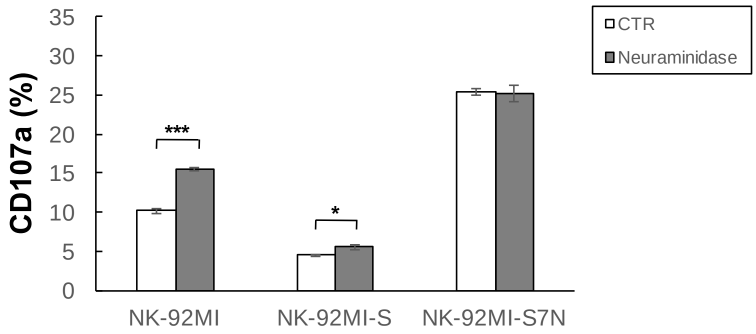

Figure S1. Cell surface sialylation on target cells influenced NK degranulation activity of NK-92MI and NK-92MI-S cells. CD107a degranulation activity in NK-92MI, -S, and -S7N were examined by incubations of neuraminidase-treated and untreated control THP-1 cells with equal number of fluorescence-labeled NK cells. Results were presented as mean ± SD of triplicates (**P*<0.05; *** *P*<0.001). The results were representative of three independent experiments.
